# Variation in Responses of Photosynthesis and Apparent Rubisco Kinetics to Temperature in Three Soybean Cultivars

**DOI:** 10.3390/plants8110443

**Published:** 2019-10-23

**Authors:** James Bunce

**Affiliations:** Adaptive Cropping Systems Lab (Retired), USDA-ARS, Beltsville, MD 20705, USA; buncejames49@gmail.com; Tel.: +1-410-451-7343

**Keywords:** photosynthesis, Rubisco, temperature, acclimation, soybean

## Abstract

Recent in vivo assays of the responses of Rubisco to temperature in C_3_ plants have revealed substantial diversity. Three cultivars of soybean (*Glycine max* L. Merr.), Holt, Fiskeby V, and Spencer, were grown in indoor chambers at 15, 20, and 25 °C. Leaf photosynthesis was measured over the range of 15 to 30 °C, deliberately avoiding higher temperatures which may cause deactivation of Rubisco, in order to test for differences in temperature responses of photosynthesis, and to investigate in vivo Rubisco kinetic characteristics responsible for any differences observed. The three cultivars differed in the optimum temperature for photosynthesis (from 15 to 30 °C) at 400 μmol mol^−1^ external CO_2_ concentration when grown at 15 °C, and in the shapes of the response curves when grown at 25 °C. The apparent activation energy of the maximum carboxylation rate of Rubisco differed substantially between cultivars at all growth temperatures, as well as changing with growth temperature in two of the cultivars. The activation energy ranged from 58 to 84 kJ mol^−1^, compared with the value of 64 kJ mol^−1^ used in many photosynthesis models. Much less variation in temperature responses occurred in photosynthesis measured at nearly saturating CO_2_ levels, suggesting more diversity in Rubisco than in electron transport thermal properties among these soybean cultivars.

## 1. Introduction

The temperatures at which crop leaves accomplish photosynthesis vary diurnally, seasonally, and with geographic location. Intraspecific variation in the response and acclimation of photosynthesis to temperature has been studied extensively since the first infra-red CO_2_ analyzers came into use in plant physiology [1,2]. Photosynthetic response and acclimation to temperature has often been studied in crop species, such as barley, broad bean, soybean, sunflower, tomato, and turnip [3]. Intraspecific variation in responses to brief, extreme temperature events has also been documented in maize, soybean, tomato and wheat [4,5,6,7]. However, studies of intraspecific variation in photosynthetic response or acclimation to temperature in crops are rare. Intraspecific variation in response or acclimation of photosynthesis to temperature could prove a useful avenue for crop improvement or for the matching of plant physiological characteristics with climate. That strategy may avoid the inverse relationship between leaf size and photosynthetic rate commonly found in studies of intraspecific variation in photosynthesis in crops, which limits the usefulness of photosynthetic rate itself as a selection criterion [8].

Photosynthesis of C_3_ species measured at high light and at the current atmospheric CO_2_ concentration has an optimum temperature which may vary with species, and sometimes with growth temperature [9,10,11]. Growth at different temperatures may also affect the maximum photosynthetic rates without changing the shape of the temperature response curves or the optimal temperature [3,10,11]. Several recent studies have found considerable variation in the kinetic properties of Rubisco which affected the response of photosynthesis to temperature [10,12,13,14,15,16], but none of these studies compared cultivars of soybean. 

Soybean (*Glycine max* L. Merr.), while of sub-tropical origin, is grown in North America from southern Canada to the gulf coast states of the southern United States of America. Mean monthly temperatures during soybean growing seasons range from about 15 °C in the north to about 27 °C in the south, while midday mean temperatures during the growing seasons range from a low of about 20 °C in the early season in the north to about 32 °C in mid-season in the southern part of this range. Thus, soybeans grown in North America are subject to a wide range of both growth and midday temperatures, even without considering extreme temperature events. 

This study examined photosynthetic responses to temperature in three cultivars of soybean when grown at three growth temperatures in order to determine whether significant variation in response exists within soybeans, and to identify which kinetic parameters may be involved in any such variation. The hypothesis was that there would be insignificant variation in photosynthetic response to moderate temperatures among these soybean cultivars.

## 2. Results

**Figure 1:**
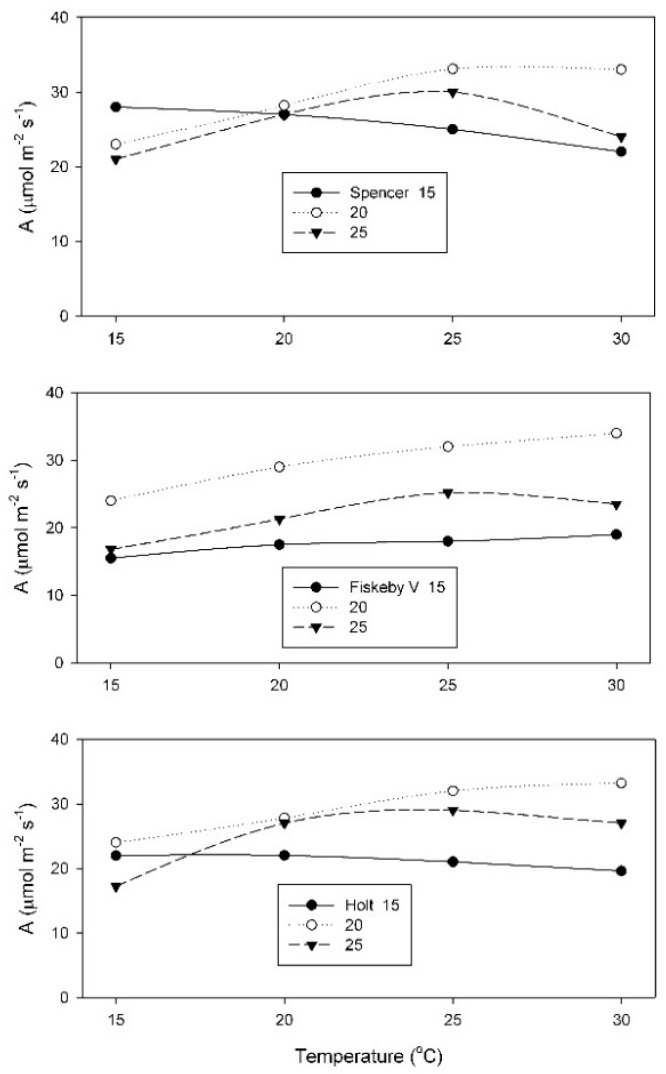
Values of the net rate of CO_2_ assimilation (A) measured at 400 μmol mol^−1^ external CO_2_ over a range of leaf temperatures, for plants of three cultivars, grown at 15, 20, or 25 °C. Each point represents a mean for 3 or 4 leaves.

The net rate of CO_2_ assimilation (A) measured at 400 μmol mol^−1^ CO_2_ was highest at temperatures which ranged from 15 to 30 °C, depending upon the cultivar and the growth temperature (Figure 1). The 20 °C growth temperature produced leaves with the highest A, for measurement temperatures of 25 and 30 °C, for all three cultivars. The shape of the response of A to measurement temperature was least affected by growth temperature in Fiskeby V, and most affected in Spencer (Figure 1). Only Spencer had highest A at 15 °C when grown at 15 °C. For the other two cultivars, highest A at 15 °C occurred in leaves grown at 20 °C. 

**Figure 2:**
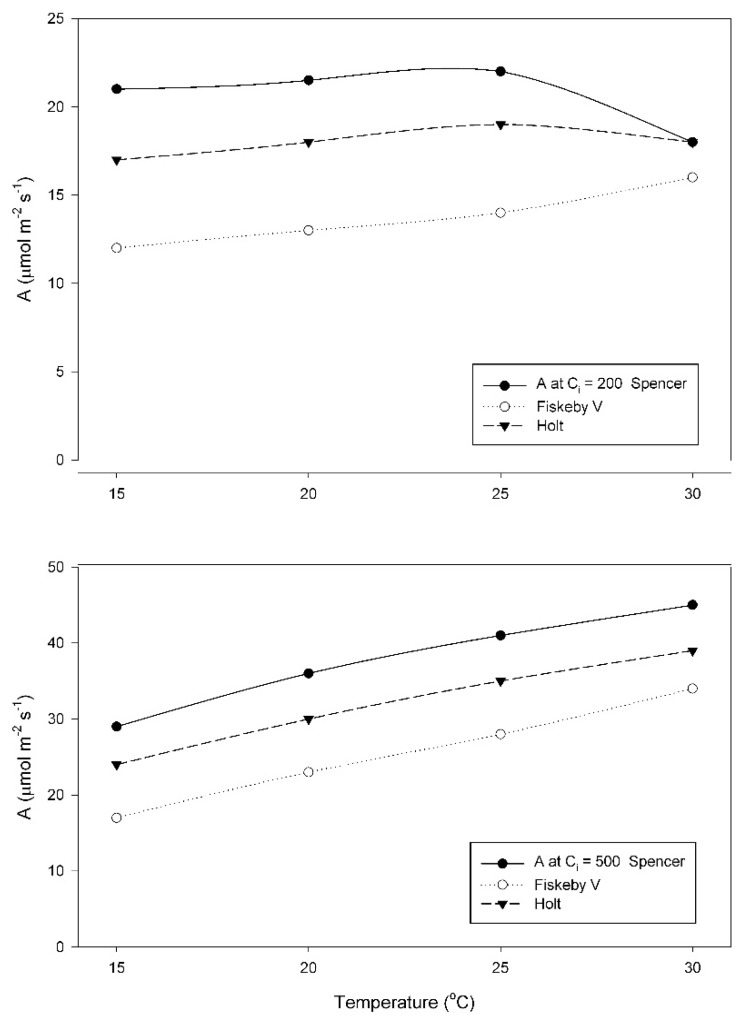
Values of A measured at either sub-stomatal CO_2_ concentration (C_i_) = 200 μmol mol^−1^, or at C_i_ = 500 μmol mol^–1^ CO_2_ for three soybean cultivars grown at 15 °C. Each point represents a mean for 3 or 4 leaves. Statistical comparisons are given in Table A1 and Table A2.

When grown at 15 °C, A at a sub-stomatal CO_2_ concentration (C_i_) of 200 μmol mol^−1^, increased between 25 and 30 °C in Fiskeby V, and decreased between 25 and 30 °C in the other two cultivars (Figure 2). Two-way ANOVA indicated significant effects of cultivar, temperature and their interaction (Table A1). In contrast, values of A obtained at a measurement C_i_ of 500 μmol mol^−1^ increased from 15 to 30 °C in all three cultivars (Figure 2). Effects of cultivar and temperature were significant, but the interaction term was not significant (Table A2). For all measurement CO_2_ conditions, A was highest in Spencer and lowest in Fiskeby V at all temperatures, with Holt intermediate. 

**Figure 3:**
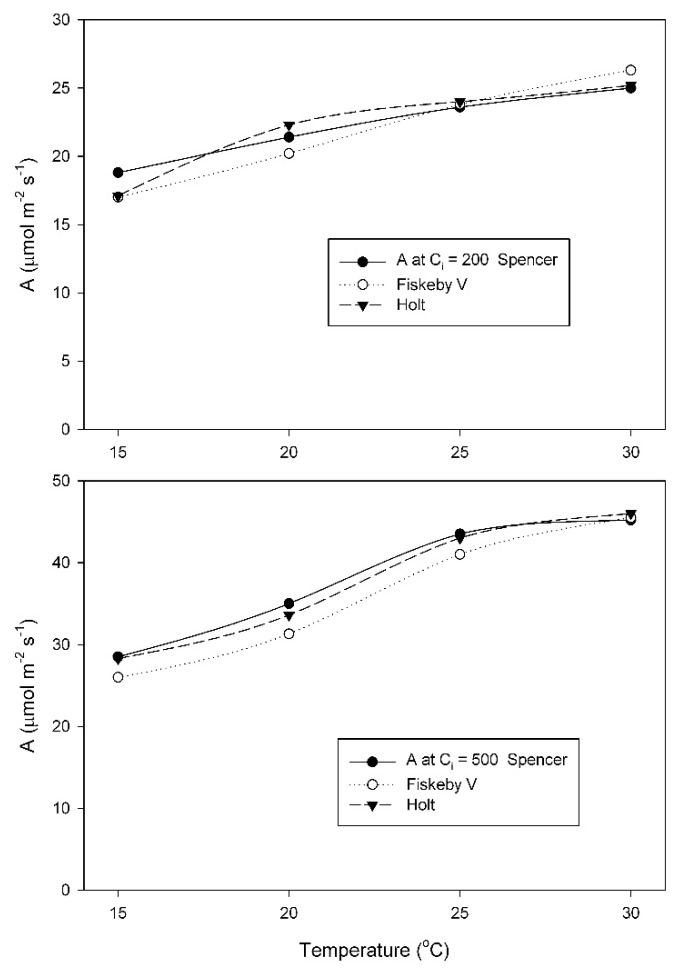
Values of A measured at either C_i_ = 200 μmol mol^−1^, or at C_i_ = 500 μmol mol^−1^ CO_2_ for three soybean cultivars grown at 20 °C. Each point represents a mean for 3 or 4 leaves. Statistical comparisons are given in Table A3 and Table A4.

When grown at 20 °C, A did not differ between cultivars at any temperature or measurement CO_2_ condition, and increased between 15 and 30 °C (Figure 3). For each measurement CO_2_ condition, two-way analysis of variance indicated a significant effect of measurement temperature, but no cultivar effects and no interaction effects (Table A3 and Table A4). 

**Figure 4:**
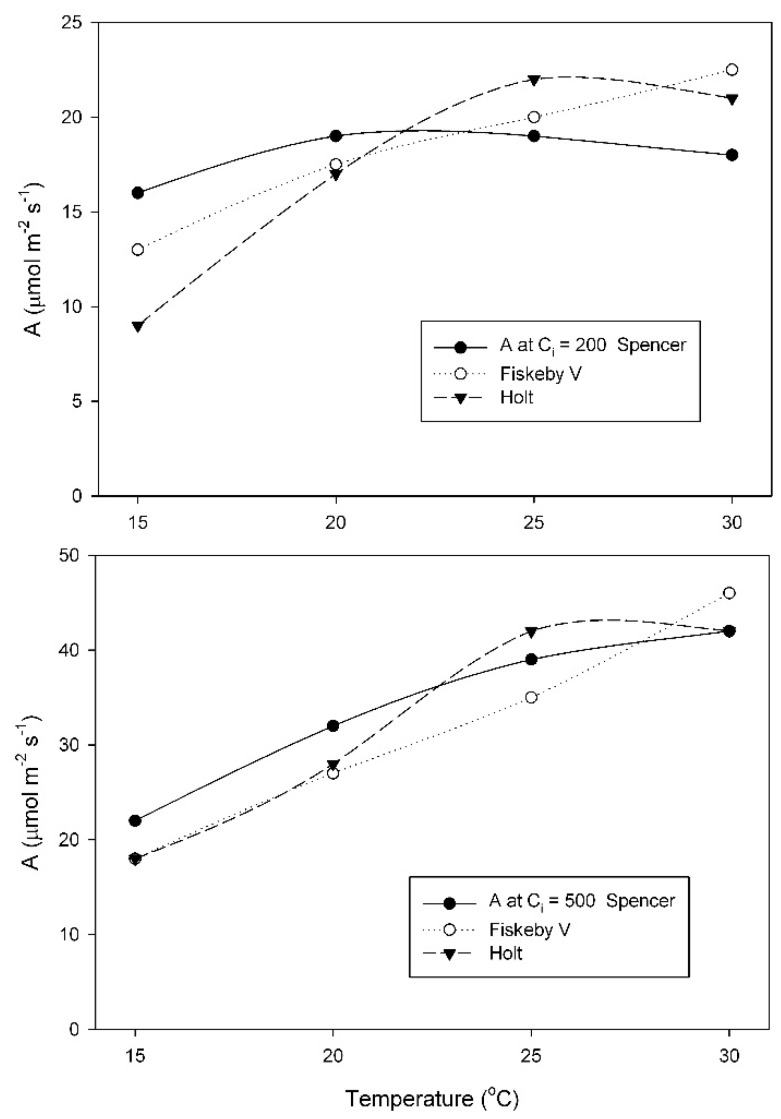
Values of A measured at either C_i_ = 200 μmol mol^−1^, or at C_i_ = 500 μmol mol^−1^ CO_2_ for three soybean cultivars grown at 25 °C. Each point represents a mean for 3 or 4 leaves. Statistical comparisons are given in Table A4 and Table A6.

At the growth temperature of 25 °C, A at the measurement C_i_ = 200 μmol mol^−1^ changed much less with temperature in Spencer than in Holt or Fiskeby V (Figure 4). For measurements at C_i_ = 200 μmol mol^−1^, effects of cultivar, temperature, and their interaction were all significant (Table A5). At 500 μmol mol^−1^ measurement C_i_, all three cultivars had similar increases in A with temperature (Figure 4), with only the effect of temperature being significant (Table A6).

Mesophyll conductance did not vary substantially with either cultivar or growth temperature, but increased strongly with measurement temperature (Table 1). Because mesophyll conductance did not vary among cultivars or with growth temperature, the values of respiration in the light (R_l_) and CO_2_ concentration at which carboxylation equals photorespiratory CO_2_ release (Γ*), which were only used for the calculation of mesophyll conductance, are not shown.

**Figure 5:**
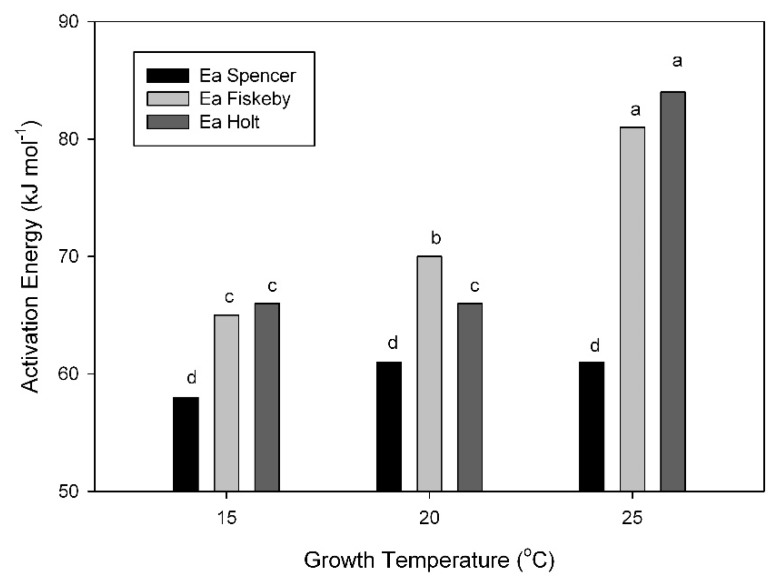
The activation energy (Ea) of the maximum carboxylation capacity (V_Cmax_) of Rubisco based on C_i_, for three cultivars of soybean grown at three temperatures (15, 20, or 25 °C). Each column represents a mean for 3 or 4 leaves. Different letters indicate significant differences at *p* = 0.05, using analysis of variance.

The activation energy of the maximum carboxylation capacity of Rubisco (V_Cmax_) was, in all cases, lower by 2 to 3 kJ mol^−1^ when based on the CO_2_ concentration at Rubisco (C_c_) compared with C_i_. The activation energy of V_Cmax_ based on C_i_ was consistently lower in Spencer than in the other cultivars (Figure 5). The activation energy did not change substantially with growth temperature in Spencer, but increased at growth temperatures of 20 and 25 °C in Fiskeby V and Holt (Figure 5).

## 3. Discussion

This work indicated a wide range of responses of photosynthesis to growth and measurement temperature within only three commercial cultivars of soybean. Photosynthetic rates at high light and at air levels of CO_2_ varied by at least a factor of 1.8 among the three cultivars at all measurement temperatures examined (15 to 30 °C), and the optimum temperatures for photosynthesis at air levels of CO_2_ ranged from at least 15 to 30 °C.

**Figure 6:**
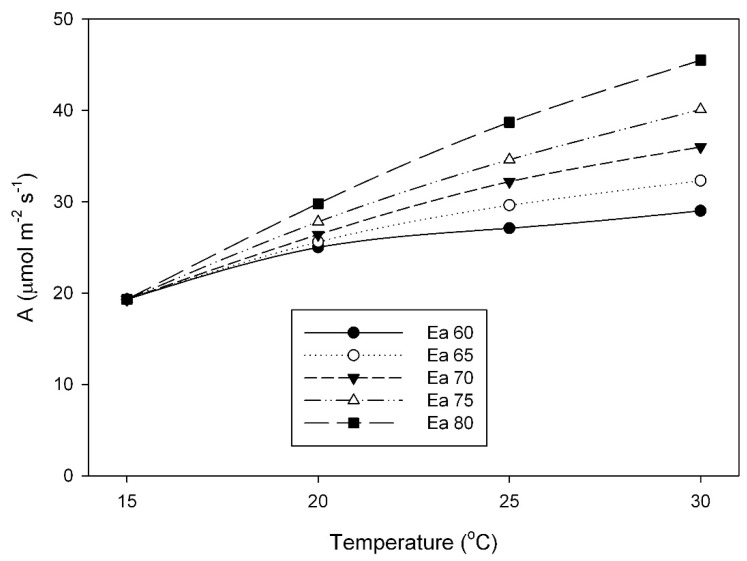
Hypothetical rates of A at the CO_2_ concentration at Rubisco (C_c_) = 250 μmol mol^–1^, for a single value of V_Cmax_ at 15 °C, combined with different activation energies (Ea, in kJ mol^–1^) of V_Cmax_ of Rubisco.

The large impact that differences in the activation energy of V_Cmax_ can have on responses of photosynthesis to temperature is illustrated in Figure 6, which shows photosynthetic rates at a C_c_ of 250 μmol mol^−1^ for a fixed value of V_Cmax_ at 15 °C, combined with different activation energies of V_Cmax_. At 30 °C, a 1.33× range in activation energy (from 60 to 80 kJ mol^–1^) would result in a 1.56× range in A. The range of activation energy values for V_Cmax_ (58 to 84 kJ mol^−1^) observed in this study is comparable to the variation among herbaceous species found by Hikoska et al. [10], and those reviewed by Kattge and Knorr [17], and also to that reported from temperature acclimation experiments with quinoa [18]. Others have also found variation in the activation energy of Rubisco among species from different climates [14], within the Triticeae [15] and within the Paniceae [16]. 

In addition to differences in the response of the V_Cmax_ of Rubisco to measurement temperature among the soybean cultivars studied here, approximately two-fold variation in the value of V_Cmax_ measured at 15 °C also occurred among these three cultivars when grown at 15 and 25 °C (Figure 2 and Figure 4). Variation in the response of V_Cmax_ to growth temperature among species and ecotypes is well known [3,19], but the possibility of intraspecific variation in crop species has received little attention to date. The cultivar Spencer had the highest photosynthetic rates of the three cultivars at air levels of CO_2_ when grown and measured at the lowest temperature, suggesting that it was well adapted to lower temperatures. The cost of this adaptation to low temperatures was presumably a larger investment in Rubisco protein per unit leaf area [9,11]. The other two cultivars could achieve as high rates of photosynthesis at high temperatures as Spencer despite presumably lower investment in Rubisco protein, because of their higher activation energies of V_Cmax_ of Rubisco. Among these soybean cultivars, intraspecific variation in the temperature dependence of V_Cmax_ was much larger than differences in the temperature dependence of maximum rates of electron transport J_max_. Consequently, the ratio of V_Cmax_ to J_max_ varied substantially among the cultivars, and with growth and measurement temperature, rather than being relatively constant, as suggested by some studies [20,21]. The temperature dependence of photosynthesis at current air levels of CO_2_ was much more closely related to V_Cmax_ than to J_max_ in these soybean cultivars, but that could change with rising atmospheric CO_2_.

Based on photosynthetic rates measured at current air levels of CO_2_, the cultivar Spencer seemed the best adapted to cool growth and measurement temperatures, as it had the highest rates of the three cultivars when grown at 15 °C and measured at 15 and 20 °C. The cultivar Holt seemed the best adapted to warm temperatures, having the highest photosynthetic rate among the three cultivars when measured at 30 °C, for plants grown at 25 °C. It may be useful to consider photosynthetic adaptation to temperature as an additional criterion for developing soybean cultivars for different locations. 

## 4. Materials and Methods 

Seeds of three cultivars of soybean (*Glycine max* L. Merr.), Holt, Fiskeby V, and Spencer were obtained from the USDA germplasm collection, and were grown in indoor, controlled environment chambers. Seeds were sown in 15 cm diameter plastic pots filled with a medium grade of vermiculite. Pots were flushed daily with a complete nutrient solution containing 14.5 mM nitrogen. Plants were grown in two M-12 chambers made by Environmental Growth Chambers (Chagrin Falls, Ohio) equipped with metal halide and high pressure sodium lamps. Twelve hours per day had light at 1000 μmol m^−2^ s^−1^ photosynthetic photon flux density (PPFD). Air temperature was controlled at 15, 20, or 25 °C, with respective dew point temperatures of 8, 13, and 19 °C. Constant temperatures were chosen in order to avoid possibly stressful low night temperatures for the low temperature treatment. All three cultivars were grown together in each chamber, with temperatures randomly assigned to chambers in successive “runs”. Each “run” had three pots of each cultivar, with one plant per pot. A total of nine “runs” were grown in order to obtain data on all of the various photosynthetic parameters. Photosynthetic characterization was accomplished using third main stem trifoliolate leaves within a few days after those leaves had reached maximum area, when tests showed that photosynthetic properties were stable over several days.

**Figure A1:**
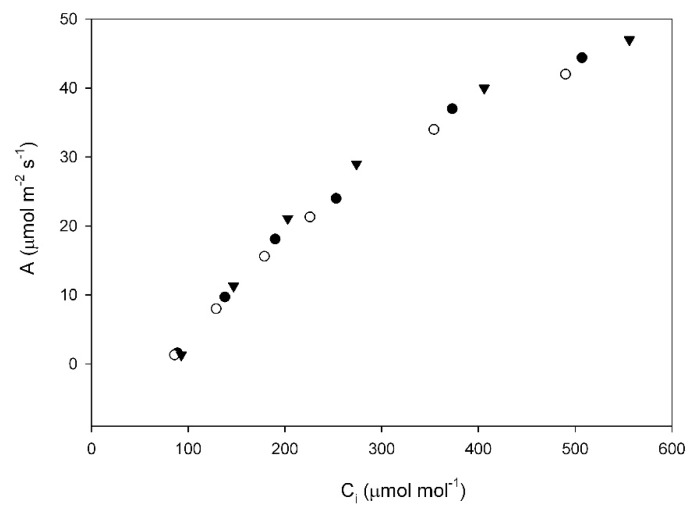
Examples of A vs. C_i_ curves. These curves are for three leaves of Spencer grown at 25 °C, and measured at 30 °C. Different symbols represent different leaves. Some data points have been omitted for clarity.

Basic responses of photosynthesis to temperature were obtained by measuring responses of A to external CO_2_ concentrations from 100 to 1200 μmol mol^−1^ at temperatures of 15, 20, 25, and 30 °C. In all cases, 1200 μmol mol^−1^ CO_2_ was saturating to A. Higher temperatures, which could lead to deactivation of Rubisco [22], were deliberately avoided. These measurements were made on three or four plants of each cultivar for each growth temperature. The small number of replicate plants was justified by the low leaf to leaf variation, as indicated by low values of residual mean squares (Table A1, Table A2, Table A3, Table A4, Table A5 and Table A6), as can also be seen in the example of an A vs. C_i_ curve in Figure A1. Gas exchange measurements were made with a CIRAS-3 portable photosynthesis system (PPSystems, Amesbury MA) operated within a controlled environment chamber. During the gas exchange measurements, leaf temperature was controlled to ± 0.3 °C, the PPFD was 1500 μmol m^−2^ s^−1^, and the leaf to air water vapor pressure deficit ranged from about 0.9 kPa at 15 °C to about 1.4 kPa at 30 °C. The temperature of the controlled environment chamber was set to match the target leaf temperature, and the chamber PPFD was 1000 μmol m^−2^ s^−1^.

Responses of A to sub-stomatal CO_2_ concentration (C_i_) were determined using either traditional steady-state measurements at external CO_2_ concentrations of 400, 100, 150, 200, 250, 300, 400, 500, 600, 800, 1000, and 1200 μmol mol^−1^ sequentially, or transient measurements during linear ramping of CO_2_ concentrations from 100 to 1200 μmol mol^−1^ [23]. The CO_2_ ramping technique compares apparent CO_2_ fluxes for an empty chamber with those when a leaf is present to obtain values of A at approximately 6 μmol mol^−1^ CO_2_ intervals. Because stomatal conductance did not change during the CO_2_ ramping, values of C_i_ could be calculated for each value of A. Details of the CO_2_ ramping method using the CIRAS-3 instrument, and examples comparing A vs. C_i_ curves obtained by ramping and by steady-state measurements are given in Bunce (2018) [23]. The advantage of the CO_2_ ramping method is that a complete A vs. C_i_ curve could be obtained in about 5 min, compared with about 30 min for a steady-state response curve. For each cultivar, growth temperature, and measurement temperature, comparisons were made of A vs. C_i_ curves obtained on the same leaf by the two methods to verify that photosynthetic parameters obtained by both methods did not differ substantially in this experiment.

Mesophyll conductance (g_m_) for CO_2_ movement from intercellular airspace to the site of fixation was measured for each growth and measurement temperature in all cultivars. Mesophyll conductance was determined from the oxygen sensitivity of photosynthesis in the Rubisco-limited region [24]. Because that method of measuring g_m_ depends upon knowing values of respiration in the light (R_l_) and the CO_2_ concentration at which carboxylation equals photorespiratory CO_2_ release (Γ*), R_l_ and Γ* were also measured. Γ* was measured from the intersection of A vs. C_i_ curves at high and low PPFD, using the precautions detailed by Walker and Ort (2015) [25]. R_l_ was determined by extrapolating A vs. C_i_ curves measured at 2% O_2_ to zero C_i._ The values of R_l_ and Γ* measured for each leaf were then used to calculate g_m_ by the oxygen sensitivity method [24]. Prior work in soybean indicated that g_m_ did not vary with C_i_ [26]. The CO_2_ concentration at Rubisco (C_c_) was then calculated from A and C_i_, using C_c_ = C_i_ – A/g_m_ for each set of values of A and C_i_. 

The maximum carboxylation capacity of Rubisco (V_Cmax_) was then estimated from the initial slopes of A vs. C_i_ and A vs. C_c_ curves for each leaf, growth temperature, and measurement temperature, using the temperature response functions of Bernacchi et al. [27]. The temperature dependencies of V_Cmax_ based on C_i_ and C_c_ were summarized by their activation energies over the range of 15 to 30 °C. Activation energy was calculated as the slope of 1/V_Cmax_ vs. 1/T (in °K). No deactivation term was used, since high temperatures causing deactivation were not used in this study.

Responses of photosynthesis to CO_2_ for each cultivar, growth temperature, and measurement temperature were summarized as A at an external CO_2_ (C_a_) of 400 μmol mol^−1^, which is approximately the current atmospheric CO_2_, A at C_i_ = 200 μmol mol^−1^ as an indication of V_Cmax_, and A at C_i_ = 500 μmol mol^−1^, as an indication of the electron transport-limited A (J_max_). These parameters were calculated for each leaf from the A vs. C_i_ response curves, and two-way ANOVA was used to test for effects of cultivar, temperature, and their interaction separately for the three growth temperatures, for each photosynthetic parameter.

## Figures and Tables

**Table 1:** Mesophyll conductance to CO_2_ movement from the intercellular airspace to the site of fixation (g_m_) in three soybean cultivars grown at three temperatures, and measured over the range of 15 to 30 °C. Values are means for 3 or 4 leaves. Values followed by different letters are different at *p* = 0.05, using analysis of variance on log transformed data in order make variances homogeneous.

Cultivar	Growth Temperature (°C)	Mesophyll Conductance (mol m^−2^ s^−1^)			
		Measurement Temperature (°C)			
		15	20	25	30
Holt	15	0.40d	0.93c	1.6b	2.4a
Fiskeby V	15	0.30d	0.72c	1.3b	2.6a
Spencer	15	0.28d	1.10c	1.5b	2.5a
Holt	20	0.31d	0.93c	1.7b	2.4a
Fiskeby V	20	0.35d	0.77c	1.5b	2.3a
Spencer	20	0.41d	0.95c	1.8b	2.6a
Holt	25	0.38d	0.85c	1.8b	2.3a
Fiskeby V	25	0.45d	0.75c	1.7b	2.3a
Spencer	25	0.33d	0.85c	1.6b	2.5a

## Appendix A

**Table A1:** Analysis of variance for photosynthetic rates measured at 200 μmol mol^–1^ C_i_ for three soybean cultivars grown at 15 °C.

Source	Degrees of Freedom	Sum of Squares	Mean Square	F-Value	*p*-Value
Cultivar	2	278	139	196	<0.0001
Temperature	3	8.19	2.73	3.85	0.0221
C × T	6	51.6	8.60	12.2	<0.0001
Residual	24	17.0	0.708		

**Table A2:** Analysis of variance for photosynthetic rates measured at 500 μmol mol^−1^ C_i_ for three soybean cultivars grown at 15 °C.

Source	Degrees of Freedom	Sum of Squares	Mean Square	F-Value	*p*-Value
Cultivar	2	890	445	113	<0.0001
Temperature	3	1091	364	92.5	<0.0001
C × T	6	66.5	11.1	2.82	0.0320
Residual	24	94.3	3.93		

**Table A3:** Analysis of variance for photosynthetic rates measured at 200 μmol mol^−1^ C_i_ for three soybean cultivars grown at 20 °C.

Source	Degrees of Freedom	Sum of Squares	Mean Square	F-Value	*p*-Value
Cultivar	2	0.945	0.472	0.484	0.626
Temperature	3	418	139	143	<0.0001
C × T	6	7.05	1.18	1.20	0.327
Residual	36	35.2	0.976		

**Table A4:** Analysis of variance for photosynthetic rates measured at 500 μmol mol^−1^ C_i_ for three soybean cultivars grown at 20 °C.

Source	Degrees of Freedom	Sum of Squares	Mean Square	F-Value	*p*-Value
Cultivar	2	41.0	20.5	0.616	0.546
Temperature	3	2413	714.6	21.5	<0.0001
C × T	6	186	30.9	0.929	0.486
Residual	36	1198	33.3		

**Table A5:** Analysis of variance for photosynthetic rates measured at 200 μmol mol^−1^ C_i_ for three soybean cultivars grown at 25 °C.

Source	Degrees of Freedom	Sum of Squares	Mean Square	F-Value	*p*-Value
Cultivar	2	3.50	1.75	3.00	0.0687
Temperature	3	303	101	173	<0.0001
C × T	6	93.5	15.6	26.7	<0.0001
Residual	36	14.0	0.583		

**Table A6:** Analysis of variance for photosynthetic rates measured at 500 μmol mol^−1^ C_i_ for three soybean cultivars grown at 25 °C.

Source	Degrees of Freedom	Sum of Squares	Mean Square	F-Value	*p*-Value
Cultivar	2	30.5	15.3	14.1	<0.0001
Temperature	3	3068	1023	944	<0.0001
C × T	6	149.5	24.9	23.0	<0.0001
Residual	24	26.0	1.08		
